# Review of Flexible Robotic Grippers, with a Focus on Grippers Based on Magnetorheological Materials

**DOI:** 10.3390/ma17194858

**Published:** 2024-10-02

**Authors:** Meng Xu, Yang Liu, Jialei Li, Fu Xu, Xuefeng Huang, Xiaobin Yue

**Affiliations:** Institute of Machinery Manufacturing Technology, China Academy of Engineering Physics, Mianyang 621999, China; 18792786751@163.com (M.X.); lijialei2310@163.com (J.L.); xufu_hit@163.com (F.X.); m15823813766@163.com (X.H.); yuexbin@sohu.com (X.Y.)

**Keywords:** magnetorheological materials, robotic, flexible grippers, shape memory alloy, pneumatic, dielectric elastomer

## Abstract

Flexible grippers are a promising and pivotal technology for robotic grasping and manipulation tasks. Remarkably, magnetorheological (MR) materials, recognized as intelligent materials with exceptional performance, are extensively employed in flexible grippers. This review aims to provide an overview of flexible robotic grippers and highlight the application of MR materials within them, thereby fostering research and development in this field. This work begins by introducing various common types of flexible grippers, including shape memory alloys (SMAs), pneumatic flexible grippers, and dielectric elastomers, illustrating their distinctive characteristics and application domains. Additionally, it explores the development and prospects of magnetorheological materials, recognizing their significant contributions to the field. Subsequently, MR flexible grippers are categorized into three types: those with viscosity/stiffness variation capabilities, magnetic actuation systems, and adhesion mechanisms. Each category is comprehensively analyzed, specifying its unique features, advantages, and current cutting-edge applications. By undertaking an in-depth examination of diverse flexible robotic gripper types and the characteristics and application scenarios of MR materials, this paper offers a valuable reference for fellow researchers. As a result, it facilitates further advancements in this field and contributes to the provision of efficient gripping solutions for industrial automation.

## 1. Introduction

With the continuous development of industrial automation technology, labor-intensive businesses are emphasizing the use of automated equipment to replace manual labor. This change not only helps reduce labor costs but also enhances operational efficiency. One of the indicators of industrial modernization today is the use of automation equipment to complete complex, cyclical, repetitive, high-intensity work in a range of fields. Automation equipment has reached a significant stage of development and is now extensively deployed. As the final actuator of the automation equipment, the robot gripper is the crucial component to achieve the various action functions. It will directly affect the use of the equipment and performance. The conventional rigid gripper has been around for a while. It has a wide range of applications and has achieved excellent results in many industries [1,2,3,4,5].

Driven by intelligent manufacturing, flexible manufacturing is gradually taking center stage in the industrial sector. Robotic systems play a crucial role in enabling flexible production. The robotic hand, serving as the end-effector for gripping target objects, holds paramount importance in determining the level of intelligent operation achieved [6,7,8]. To minimize the impact and potential damage caused by rigid contact with the target, the traditional equipment manufacturing sector frequently employs rigid manipulators outfitted with two or more fingers for grasping the target object. Additionally, precise positioning of the target part is mandated for the hand grippers. However, this type of gripping significantly amplifies the complexity of the robot. Furthermore, these rigid manipulators are typically larger in size and weight, with higher power consumption during manipulator operation [9]. On the contrary, flexible robotic grippers offer greater practicality and usability in this context.

The flexible manipulator grip is an important area of robotics. It relies primarily on the flexibility and adaptability of its own material or structure to achieve the gripping of objects. Compared to conventional rigid robotic grippers, flexible manipulators offer several advantages, including reduced weight, compact size, enhanced speed, low energy consumption, and heightened adaptability. Furthermore, they exhibit superior capability in conforming to diverse object shapes without causing damage. The distinguishing feature of flexible manipulators lies in their efficient accommodation of target parts with varying shapes, enabling precise control over gripping forces. For example, in agriculture, grippers for harvesting require pneumatic flexible grippers for flexibility and adaptability. In the medical field, due to the need for miniaturization, manipulators for drug transport can use flexible grippers based on dielectric elastomer.

There are various materials and methods currently used to manufacture flexible robotic grippers, such as shape memory alloys (SMA) [10,11,12], Pneumatic flexible grippers [13,14,15,16,17,18,19], dielectric elastomers [20,21,22,23], and magnetorheological (MR) materials [24,25,26,27,28,29,30], as shown in Table 1. The different materials and methods have their characteristics as well as potential application areas. In recent years, the review on flexible robotic grippers has not been updated, especially with regard to the application of MR materials. 

MR materials are intelligent materials whose rheological properties can be significantly altered by the stimulation of a magnetic field. This change is rapid, continuous, and reversible. Due to this property, flexible robot grippers using magnetorheological materials as a medium are one of the important directions in the development of the final actuator. The yield stress, response time, settling stability, and other properties of MR materials can directly affect the performance of flexible robot grippers. Magnetorheological fluids (MRFs) have gained traction in engineering due to their excellent magnetically controllable intelligence. These fluids have been utilized for various purposes, such as magnetorheological dampers in automotive seat suspensions [31,32,33,34], brakes [35,36,37,38], clutches [39,40,41,42], and bridges [43,44,45,46]. However, the issue of settling stability has posed a challenge to the widespread commercial adoption of magnetorheological fluids [47,48]. Researchers have worked to improve the settling stability of MR materials to address this issue. One approach involves modifying the viscosity of the matrix of the MR material, utilizing substances such as grease, silicone, and silicone rubber, among others. This pursuit has led to the development of other magnetorheological material variants, including magnetorheological grease (MRG), magnetorheological gels (MRGs), magnetorheological plastomer (MRP), and magnetorheological elastomer (MRE) [49,50,51,52].

**Table 1:** Flexible robotic grippers for different materials and methods [10,20,25,53].

Different materials and methods	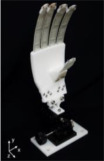	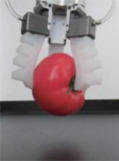	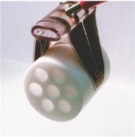	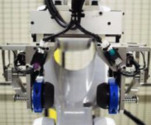
	SMA	Pneumatic flexible gripper	Dielectric elastomers	MR materials
Material properties	Shape memory effect, Superelasticity, Wear resistant	Higher output forces, Fast response time, Low cost	Low power consumption, Fast response time, Small volume	Higher output forces, Good controllability, Low cost
Material disadvantage	High energy consumption, Unstable response time, Limited lifespan	High energy consumption, Large size, Loud noise	Low output forces, Poor stability, Limited lifespan	High sealing requirements

This paper presents a comprehensive review encompassing the principles, structural design, control strategies, and performance characteristics of diverse types of flexible robotic grippers, along with an exploration of their progress in practical applications. In addition, it focuses on the material characteristics of various MR materials and their current research status in flexible robotic grippers. Finally, the working prospects of these flexible robotic grippers are summarized and discussed.

## 2. Flexible Robotic Gripper

The flexible gripper represents a novel end-effector design constructed using flexible materials. By the inherent adaptability of the smart materials, these grippers deliver several attributes, including heightened flexibility, robust environmental adaptability, and enhanced safety in human-machine interactions. It can securely grasp objects of varying shapes without causing any damage. This paper categorizes flexible robotic grippers into three distinct types based on the materials employed in their manufacturing: shape memory alloys, pneumatic fingers, and dielectric elastomers. Differences in these materials significantly influence the structural dimensions and gripping performance of the flexible robotic grippers, leading to distinct disparities among them. 

### 2.1. Shape Memory Alloy (SMA)

Shape memory alloys (SMA) belong to a class of intelligent materials renowned for their remarkable shape memory effect [54]. By harnessing this effect, SMAs generate forces and displacements upon heating. Thanks to its uncomplicated structure and impressive energy density, SMA can be fabricated into wires or springs, enabling their application as actuators in various domains, including robotics, medical instruments, and aerospace [55,56,57,58].

**Figure 1:**
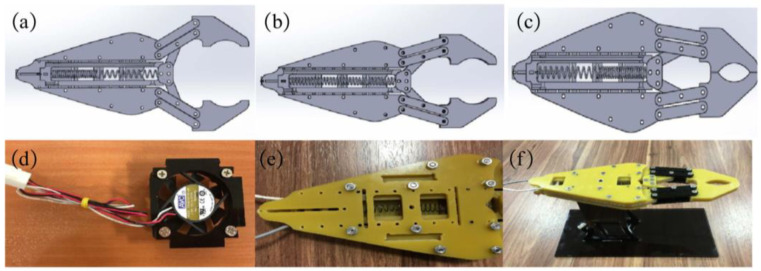
A flexible manipulator driven by SMA springs and its operating position of SMA gripper. (**a**) Open, (**b**) Transition, (**c**) Close, (**d**) Embedded fan, (**e**) Air inlets, (**f**) Final fabricated gripper [59].

SMA exhibits numerous exceptional properties, such as a high return strain, low operational noise, and excellent biocompatibility. Consequently, it finds widespread utility in flexible robotic grippers, among other fields. Rad N F et al. designed and fabricated a flexible manipulator driven by SMA springs, as shown in Figure 1a [59]. The closing and opening of the grippers are controlled by an SMA spring actuated by an applied voltage, as shown in Figure 1a–c. The use of the SMA spring in the gripper reduces costs and makes it easier to manufacture. A fan is also installed in the gripper to facilitate the low operating frequency of the SMA spring, as shown in Figure 1e. Furthermore, due to the miniature size of the SMA springs and their ability to undergo small deformation rates, these grippers can be effectively deployed in microsystems. This adaptability enables the handling of delicate micro-components and renders them particularly suitable for deployment in micro-robotic devices, such as surgical fixtures.

**Figure 2:**
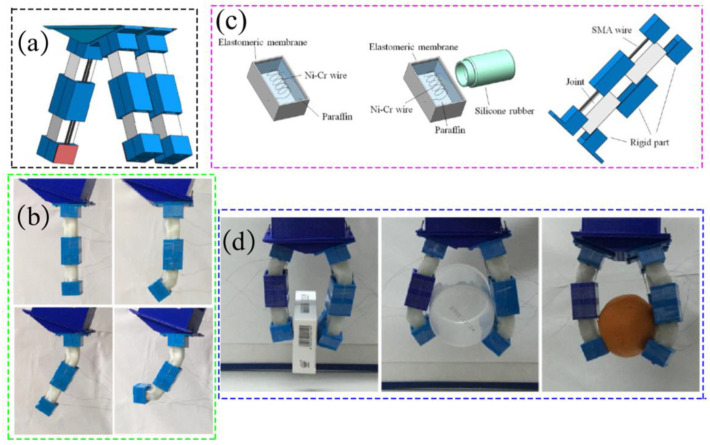
An SMA-based flexible gripper with variable stiffness. (**a**) Schematic diagram of grippers, (**b**) Each robot finger can maintain four configurations, (**c**) Embedding nickel-chromium wire and assembling, (**d**) Grabbing different objects [60].

Liu M F et al. proposed a SMA-based hand gripper with variable stiffness, as shown in Figure 2a [60]. Each gripper consists of three fingers, and each finger with two joints can actively achieve multiple postures by varying the corresponding stiffness of the joints and actuating SMA wires, as shown in Figure 2b. By incorporating paraffin wax into the rigid component, it becomes possible to achieve a variable stiffness range for the hand claw, which can be controlled by heating and cooling the paraffin wax. This mechanism is illustrated in Figure 2c. Experimental findings indicate that a single robotic finger can achieve a substantial 18-fold increase in stiffness when grasping objects, as depicted in Figure 2d.

As can be seen, robotic grippers made from the shape memory alloy type have many advantages, such as tiny structure, low cost, easy manufacture, and high gripping force. As a result, SMA robotic grippers have a wide range of applications in the fields of healthcare and soft micro-robotics. However, shape memory alloys have the disadvantages of fatigue, and long and unpredictable phase transition times, which make it difficult to ensure good reliability during the action of the gripper [61,62,63,64], as shown in Table 1.

### 2.2. Pneumatic Flexible Gripper

Pneumatic flexible grippers are constructed using materials that possess flexibility or retractability. As a result, these grippers inherently offer adaptability and can be deformed to effectively conform to the shape of the targeted object, thereby enabling the secure handling of objects with varying sizes while ensuring high levels of flexibility and safety [14]. The pneumatic flexible gripper boasts a broad range of applications and finds suitability in fields such as food processing, automotive, daily chemical, medical, 3C electronics, and others. These grippers can be integrated into intelligent assembly systems, automatic sorting processes, logistics and storage operations, and food processing lines. Additionally, they can serve as functional accessories in scientific research and experimental equipment, intelligent entertainment devices, or service-oriented robots [13,17,65].

Research on pneumatic soft hand claws first began in 1990. SUZUMO R I designed a three-degree-of-freedom soft-body actuator and analyzed its kinematic characteristics using finite element analysis and characteristic equation methods [66]. In recent years, researchers have started to apply pneumatic fingers to practical scenarios such as agriculture and industry.

**Figure 3:**
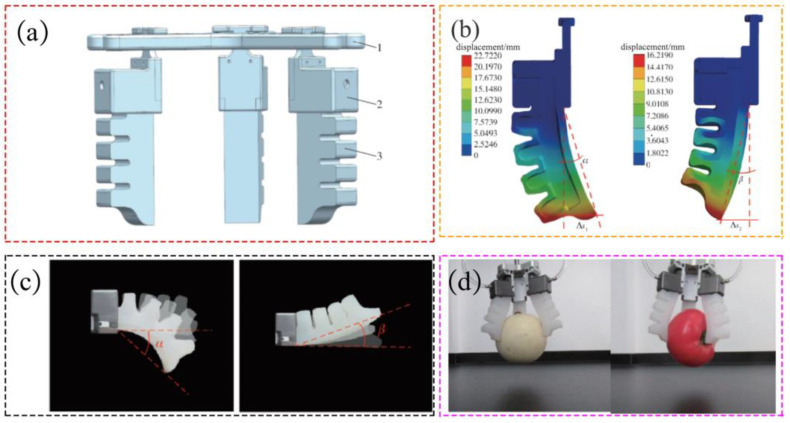
A pneumatic flexible gripper for fruit picking. (**a**) Schematic diagram of flexible gripper structure (1. Base. 2. Fixed end. 3. Flexible finger.), (**b**) Finite element simulations of deformation, (**c**) Finger bending deformation, (**d**) Grabbing different objects [67].

Based on the structural characteristics of octopus tentacles, PI Jie et al. utilized bionic principles and additive manufacturing technology to conceive and fabricate a pneumatic flexible gripper for fruit picking [67]. The gripper exhibits a simplified design with self-adaptability, comprising three flexible fingers and fixed components, as depicted in Figure 3a. Through finite element simulation, the maximum bending angle of the gripper was determined in both the inflated and suction states, thereby establishing its operational range, as illustrated in Figure 3b,c. Experimental assessments conducted on the gripping force of the flexible manipulators revealed their capability to adaptively grasp fruits weighing 564 g and measuring 100 mm in diameter within a pressure range of 0~100 kPa. Importantly, the fruits exhibited no surface damage during the gripping process, highlighting the gripper’s effectiveness, as depicted in Figure 3d.

**Figure 4:**
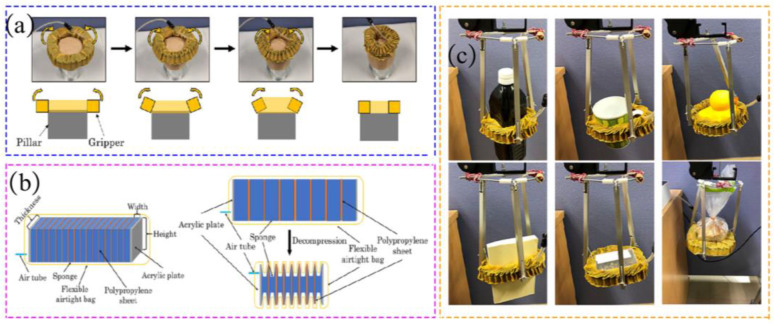
A ring-shaped pneumatic gripper. (**a**) The working principle of the gripper, (**b**) Working principle of telescopic actuator, (**c**) Grabbing different objects [68].

An alternative design for a pneumatic gripper in the form of a ring was proposed by Yamada K et al. [68]. This gripper exhibits a unique functionality where it can be wrapped around an object similar to a rubber band, as depicted in Figure 4a. The gripper comprises hoses that enclose laminated sponges interwoven with plastic sheets. When activated, as shown in Figure 4b, the air within the sponges is expelled, causing them to contract and reduce the diameter of the ring. As a result, the gripper securely holds the object, ensuring a firm grip, as depicted in Figure 4c.

From the perspective of current applications, pneumatic flexible grippers have the advantages of high reliability, low cost, and good gripping effect, making them widely utilized in the agricultural sector. However, the existing pneumatic flexible grippers still possess certain limitations that hinder their broader adoption in robotics. These drawbacks include a limited gripping angle range, large structural size, extended pneumatic response time, and high power demands for operation. These inherent deficiencies impede the gripper’s application potential.

### 2.3. Dielectric Elastomer

Dielectric elastomer is a group of intelligent soft materials. In response to an external electric field stimulus, it can generate substantial forces and undergo apparent shape and volume changes. It has a low modulus, high deformability, low noise, low density, quick response, efficient electro-force conversion, high energy density, and a high dielectric constant, among other benefits [69,70,71]. These mechanical properties, such as a low modulus of elasticity and extensive strain capacity, enable effective control of dielectric elastomers through the application of voltage. Widely recognized as “artificial muscles”, dielectric elastomer holds substantial potential for diverse applications, notably in flexible actuators, energy harvesting, flexible sensors, vibration control, and loudspeakers [72,73,74].

**Figure 5:**
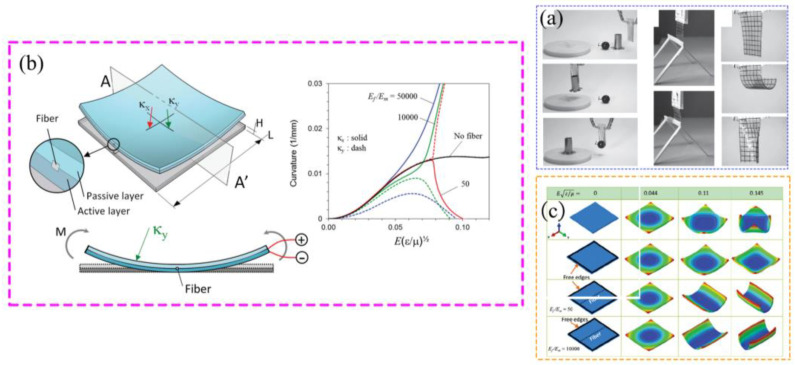
A flexible manipulator based on a dielectric elastomer actuator. (**a**) The overall structure, (**b**) Aluminum foil connecting the electrode to a power source, (**c**) Finite element simulation results of the electrode [75].

Shian S. et al. designed a flexible manipulator based on dielectric elastomer actuators [75], as shown in Figure 5a. The manipulator is based on combining several rigid fibers to guide the deformation to achieve adaptation to a variety of complex contact surfaces. They cut acrylic elastomers into thin sheets and then used Teflon filters to deposit carbon nanotube electrodes on both sides of the sheet. The electrode sheet is then bonded to another sheet of acrylic elastomer to form a double layer. Aluminum foil was used to connect the carbon nanotube electrodes to the power cable, as shown in Figure 5b. Finite element simulations of the mechanical gripper as a whole were carried out to test the deformation of the electrodes under different loading conditions, as shown in Figure 5c. The overall structure of this flexible manipulator is simple but has a large area of action and is suitable for gripping small masses of complex shapes.

**Figure 6:**
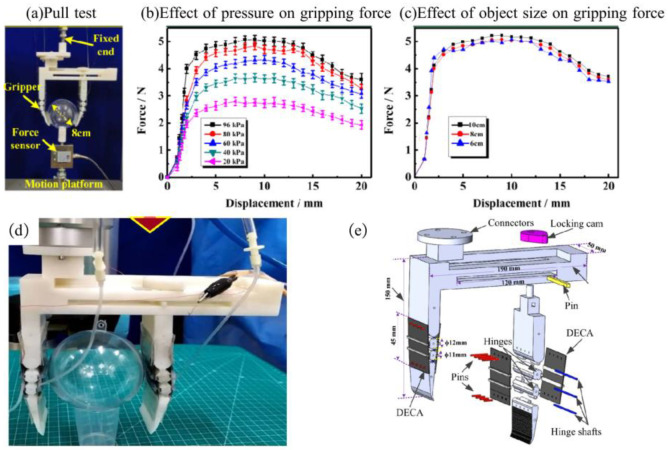
A flexible gripper with adjustable stiffness based on the dielectric elastomer. (**a**) The pull test, (**b**) The effect of pressure on gripping force, (**c**) The effect of object size on gripping force, (**d**) The overall structure, (**e**) The Assembly process [76].

Liu Lei et al. have designed and fabricated a robot gripper with adjustable stiffness using a dielectric elastomer [76], as depicted in Figure 6a. This manipulator features a more intricate structure and larger dimensions compared to other grippers. The maximum size limit of the gripped object of the robotic gripper is 12 cm. And it can exert a maximum output force of 5 N, as shown in Figure 6b,c. The entire robot gripper was printed in three-dimensional printing, and the assembly process is shown in Figure 6d,e. Notably, locking cams are employed to secure one leg of the gripper, which is also removable to adjust the gripping size. The fingertips are uniquely designed with a curved structure to enhance gripping capabilities for objects.

Despite the favorable electrically induced deformation properties of dielectric elastomers, they also exhibit certain disadvantages, such as high drive voltage requirements, low energy conversion efficiency, and limited adhesion force generation. As can be seen from the above dielectric elastomer-based flexible manipulators, this type of flexible manipulator has a low gripping force and is not suitable for gripping large objects. Consequently, these limitations impede the widespread adoption of dielectric elastomer-based manipulators.

## 3. Magnetorheological Grippers

Magnetorheological (MR) materials are a class of intelligent materials that exhibit remarkable changes in their rheological properties when subjected to magnetic field stimulation. This transformation occurs rapidly, continuously, and reversibly. The unique properties of magnetorheological materials make them extremely attractive for the purpose of manipulator development. Over other robotic grippers, MR grippers offer advantages such as enhanced safety, precise control, responsiveness, and cost-effectiveness. As a result, researchers have been devoting significant attention to the exploration and advance of MR grippers.

### 3.1. Magnetorheological Materials

Magnetorheological (MR) materials belong to a category of soft materials that exhibit magnetic sensitivity. They are created by dispersing micron-sized soft magnetic particles in various carrier substances. The exceptional feature of MR materials is their ability to undergo continuous and rapid alterations in their rheological properties when subjected to a magnetic field. This unique characteristic has captured growing attention for numerous applications in industries including construction, vibration control, and automotive sectors [77].

Based on the physical state of MR materials in the absence of an applied magnetic field and the characteristics of the substrate, MR materials can be broadly categorized into magnetorheological fluids (MRFs), magnetorheological elastomers (MREs), magnetorheological greases (MRGs), and other types [52,78,79]. These different classifications of magnetorheological materials have been developed to serve various applications and address distinct material challenges. They complement one another in terms of their specific properties and cannot be fully substituted by one another. The following provides a concise overview of the development of MR materials.

**Figure 7:**
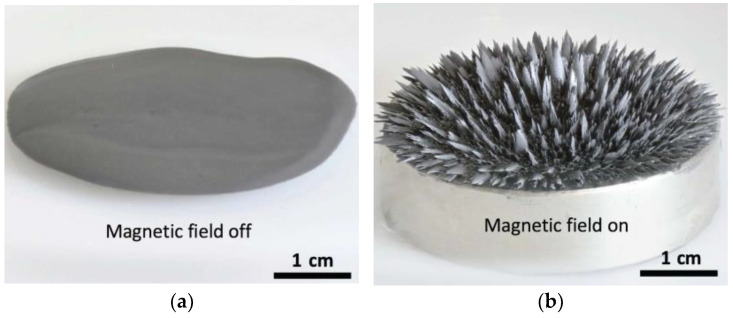

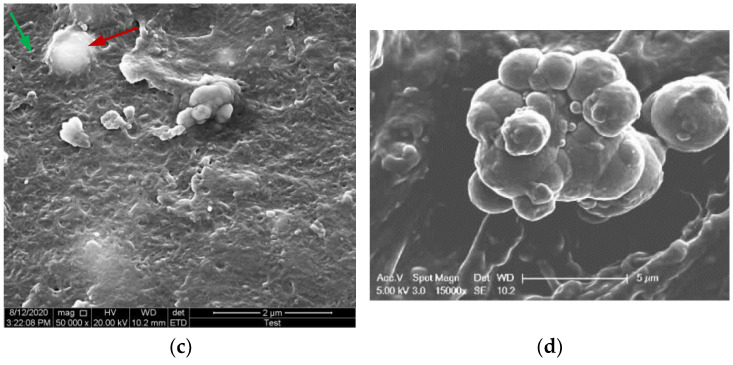
Macroscopic and microscopic properties of MR materials. (**a**) Macroscopic state of MRF in the absence of a magnetic field; (**b**) Macroscopic state of MRF in the presence of a magnetic field [80]; (**c**) Schematic of magnetic particles attached to a soap fiber structure in MRG [79]; (**d**) Schematic of ferromagnetic particles in MRE [78]. As shown in Figure 7c, the arrows point to the magnetic particles.

Magnetorheological fluids (MRFs) were the first MR materials to emerge and are mixtures of micro- or nanoscale magnetic particles homogeneously dispersed in a base carrier fluid, with a small number of additives as auxiliary components [80]. In the absence of a magnetic field, MRFs are liquid and can be viewed as Newtonian fluids, as in Figure 7a. In contrast, when a magnetic field is applied, the MRFs form blade-like and columnar bodies along the magnetic field direction of the permanent magnet. At the time, the MRF exhibits Bingham plastic fluid behavior with a yield stress, as shown in Figure 7b. It is interesting to note that the MRFs can switch between these two states at the millimeter scale by manipulating the magnetic field. At the same time, the rheological properties of the material, such as viscosity, yield stress, and energy storage modulus, are changed by several orders of magnitude.

While MRFs and their devices have undergone extensive research, a significant issue that has hindered their widespread commercial application is the settling problem caused by the substantial density difference between ferromagnetic particles and base oil [81]. Consequently, researchers have endeavored to develop alternative kinds of magnetorheological materials by manipulating the viscosity of the magnetorheological fluid matrix, such as grease and silicone rubber.

Magnetorheological grease (MRG) uses grease as a matrix and is a colloidal dispersion system consisting of magnetic particles, base oil, thickener, and additives [82]. As shown in Figure 7c, the thickening agent is dispersed in the base oil and forms a skeleton, which acts as a support and adsorption. The base oil and magnetic particles are absorbed within this structure through the action of the thickener, resulting in the formation of a paste-like magnetorheological grease. Under the three-dimensional mesh structure of the thickener (saponified fibers) in the grease, the magnetorheological grease possesses better settling stability [83]. Leveraging their excellent relative magnetorheological effects, both MRFs and MRGs provide widespread application in vibration control devices such as dampers and buffers [84,85,86].

Magnetorheological elastomers (MREs) are prepared from ferromagnetic particles dispersed in elastomeric polymers. The first MRE was prepared by the Japanese scholar Shiga T et al. using silicone resin gels as a matrix [87]. Figure 7d illustrates the microstructure of MRE, which retains the magnetic sensitization phenomenon while displaying distinct operational characteristics compared to magnetorheological fluids. The MRE, on the other hand, regulates its modulus and damping before yielding by adjusting the magnitude of the magnetic field, thereby achieving damping and stiffness regulation [88,89]. Notably, MREs have found extensive use in applications involving seismic isolation, setting them apart in terms of their intended scope of application from MRF and MRG [90,91].

### 3.2. Magnetorheological Flexible Gripper

The previous section provided an overview of the fundamental concept of MR materials, which can be categorized into liquid MRFs, MRGs, and solid MREs based on substrate morphology. The operating principle of MR flexible grippers varies depending on the morphology of the MR material matrix. Accordingly, MR manipulators can be classified into three categories based on their working principles: the viscosity/stiffness change type, the magnet-driven type, and the adhesion force type.

#### 3.2.1. The Viscosity/Stiffness Change Type

The distinctive feature of MR materials is the change in viscosity or stiffness when subjected to a magnetic field, known as the MR effect. Therefore, the most frequently employed working principle of MR materials in flexible manipulators is managing viscosity or stiffness change. MR materials can be classified into liquid and solid states based on their matrix morphology. Liquid MR materials, such as MRF and MRG, exhibit controlled viscosity change, while solid-state MRE enables controlled stiffness change. Based on their mode of action, MR flexible grippers can be classified into overall change and segmental change.

The MR material is attached to the surface of the gripper in the overall change mode. When the robotic hand claw comes into contact with the gripped object, the material conforms to the object’s surface. The application of a magnetic field allows the MR material to solidify, allowing for effective envelope gripping of the object.

**Figure 8:**
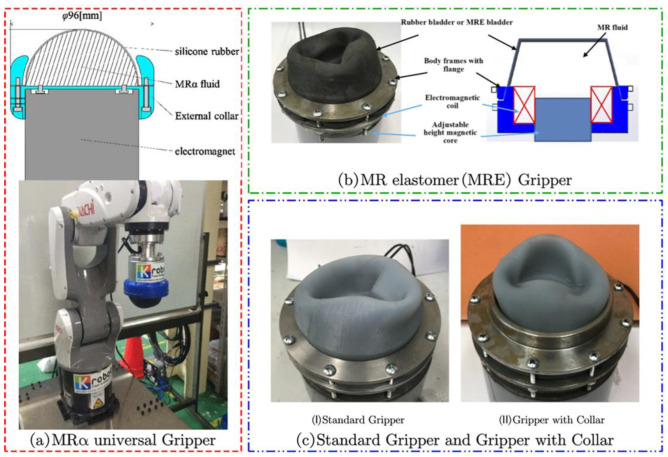
The single capsule MRF flexible gripper: (**a**) MRα universal Gripper [92], (**b**) MRE Gripper [28], (**c**) Standard Gripper and Gripper with Collar [93].

**Figure 9:**
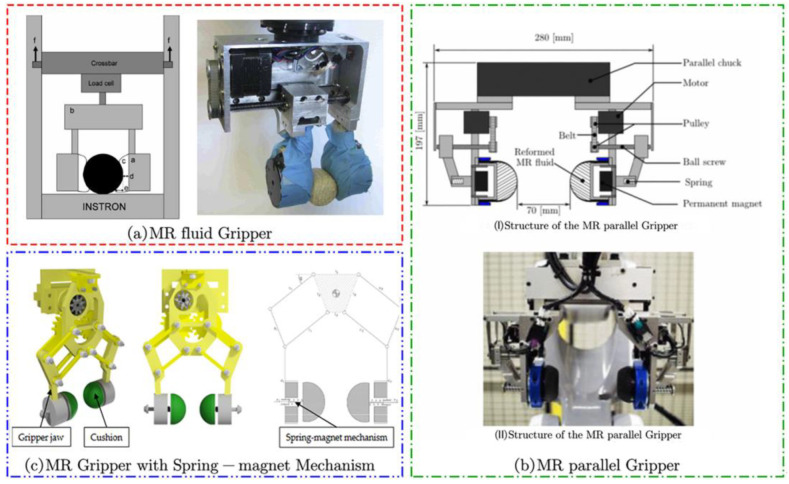
The multi-capsule type MRF flexible grippers: (**a**) MR fluid Gripper [94]; (**b**) MR parallel Gripper [25]; (**c**) MR Gripper with Spring-magnet Mechanism [95].

**Figure 10:**
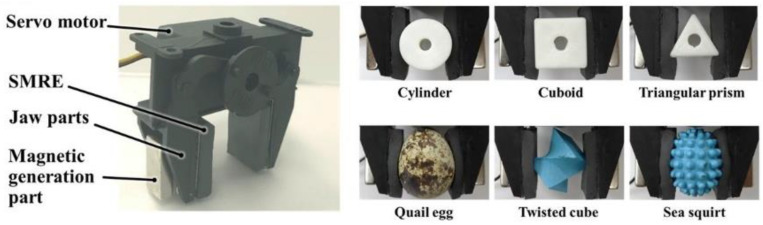
A flexible gripper with MRE skin attached [27].

Further, according to the number of grippers in contact with the object, we can classify the MR grippers that control the overall change into single capsule type (Figure 8) and multi-capsule gripping type (Figure 9 and Figure 10). The single capsule type MR gripper requires a preload force to be applied first during the gripping process so that the MR fluid capsule can better fit the surface of the object being gripped.

Okatani Y et al. of Kyushu Institute of Technology, Japan, developed a new MRα fluid by adding nonmagnetic particles to MR fluid, which can improve the curing hardness while reducing the density of MR fluid. Also, they designed a single capsule MR flexible gripper, MRα universal Gripper, using MRα fluid, and tested the flexible gripper mounted on a six-axis robot arm as shown in Figure 8a [92]. The team also investigated the effect of the diameter and mixing volume ratio of non-magnetic particles in MRα fluid on the gripping performance. The results showed that non-magnetic particles with a diameter below 0.5 mm and a mixing volume ratio of 50% with the MRF had a better gripping effect and produced a maximum gripping force of 50.67 N.

Hartzell and colleagues conducted an in-depth study on MRE capsule MR flexible grippers, as shown in Figure 8b [28] and Figure 8c [93]. They carried out a magnetic field simulation to investigate the effects of electromagnet housing shape, MRF filling, and capsule magnetism on the grasping performance of the MR flexible grippers. The outcomes demonstrated that the grasping ability of the single capsule gripper depends on the size of the preload force applied at the beginning. A higher preload force causes the object to be embedded deeper within the capsule, resulting in a more noticeable grasping effect. The researchers also contrasted the geometric properties of the grabbed targets and discovered that cylinders are simpler to grasp than spheres. Within the range of target sizes considered, the gripping force of the gripper increased as the target diameter increased.

The single capsule type MR flexible gripper has achieved a certain level of flexibility in grasping. However, it has limitations due to the large magnetic field action distance and the restricted action area of the capsule. This results in drawbacks such as a limited grasping capacity and a restricted range of target objects. In order to overcome these limitations, the researchers devised a novel approach by combining the single-capsule MR flexible grippers with the conventional rigid hand claw, leading to the development of multi-capsule MRF flexible grippers, as illustrated in Figure 9.

Based on the magnetorheological effect, Pettersson A et al. of Sweden designed the multi-capsule type MRF flexible grippers for holding and putting fruits and vegetables, as shown in Figure 9a [94]. The right side of the MRF grippers is a fixed actuator equipped with a stress-strain sensor, and the left side is a mobile hand gripper driven by a stepper motor driven by a belt drive and ball screw for moving. When the rubber capsule containing the MRF reaches the side of the object, it deforms during the gripping process to cover the object’s surface. The electromagnet in the flexible gripper is then energized to generate a magnetic field, and the yield stress of the MRF increases and transforms from a liquid to a solid state. As a result, the capsule confines the object within the MRF, enabling flexible grasping of the object. The multi-capsule type MRF flexible grippers can locate the distance between the object and the surface of the hand claw through the vision system and adjust the gap and the size of the magnetic field to obtain different grasping forces according to the size of the object.

Based on the previous MRα gripper [92], Tsugami Y et al. of Kyushu Institute of Technology, Japan, developed an MRF parallel gripper, which controls magnetic induction intensity by moving a permanent magnet, as shown in Figure 9b [25]. A servo motor and a ball screw make up the flexible gripper. The motor at the top moves the ball screw horizontally through a belt drive, which moves the permanent magnet. There is a spring incorporated into the fingertip to reduce the force of separation of the permanent magnet from the MR capsule. The flexible gripper has both “form closure” and “force closure” properties, and it regulates its grasping force by detecting the servo motor’s current. 

Białek M. et al. similarly designed MRF flexible manipulators using permanent magnets, as shown in Figure 9c [95]. This gripper changes the magnitude of the magnetic field by varying the degree of spring deformation to control the distance between the magnet and the MR capsule. They used 3D printing technology to fabricate an MR capsule of thermoplastic polyurethane and analyzed the effect of electromagnets and permanent magnets on the magnetic induction strength in the MR capsule. In addition, an experimental study was carried out to investigate the effect of MRF filling on the gripping force during pin insertion and extraction of the MRF capsule.

In contrast to MRF, which undergoes viscosity changes in response to a magnetic field, MRE exhibits changes in stiffness when subjected to a magnetic field. This characteristic eliminates the need for a sealing structure when utilizing MRE. Choi et al. conducted a study where they developed an MRE flexible manipulator by affixing MRE as a flexible skin onto the surface of a robotic hand claw, as depicted in Figure 10 [27]. Similar to the MRF flexible gripper, when the MRE flexible manipulator comes into contact with an object, the MRE material deforms based on the shape of the grasped object. Subsequently, a magnetic field is applied to solidify the MRE, enabling the grasping of the object. When the flexible gripper releases the object, the applied magnetic field is removed, and the elastomer rapidly returns to its original state.

**Figure 11:**
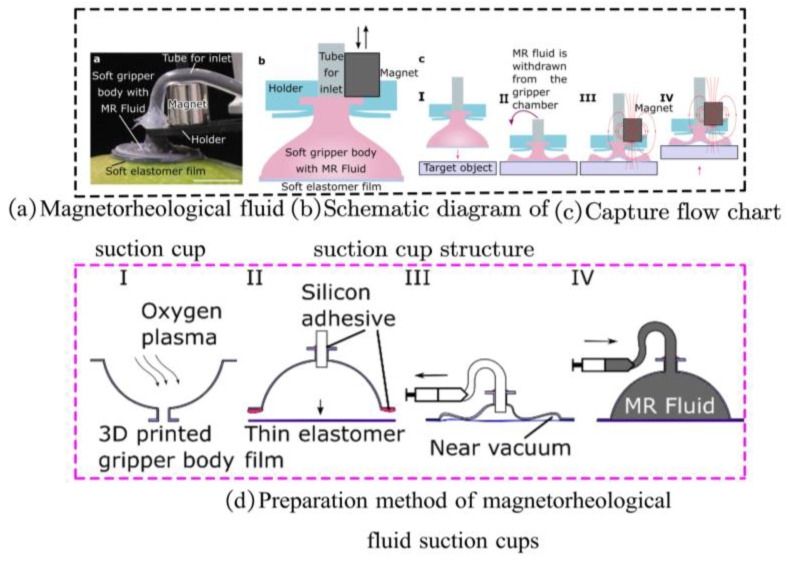
An MRF suction cup-type gripper [96].

Utilizing MRF suction cups, as shown in Figure 11, is another method for achieving flexible grasping by changing viscosity. During contact with the object, the suction cup maintains a flexible state. The MRF is expelled from the suction cup when holding a lightweight object, causing it to deform and cling to the object’s surface while still being soft. On the other hand, when gripping a heavy object, a magnetic field is applied to the suction cup, causing it to transition into a rigid state. The magnetorheological effect of the MRF contained within the suction cup facilitates this state change [96].

**Figure 12:**
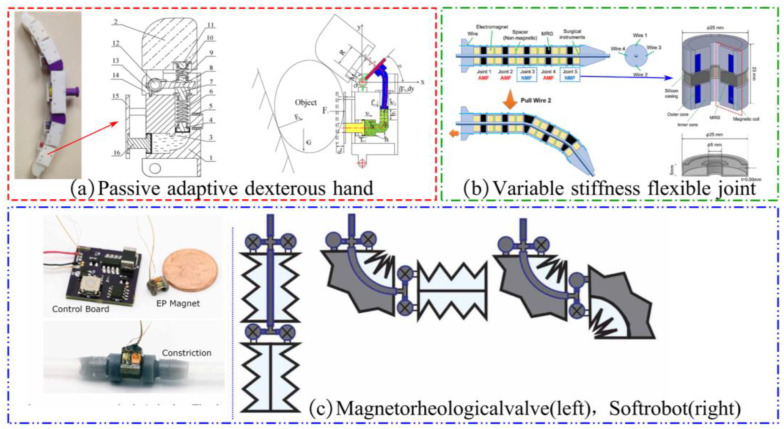
Several multi-joint magnetorheological grippers: (**a**) A passive adaptive dexterous gripper [97], (**b**) A gripper with variable stiffness and flexible joints [26], (**c**) Magnetorheological valve (left), Soft body robot (right) [98].

Unlike the previously mentioned integral viscosity-variable MR gripper, the segmented viscosity-variable gripper has MR materials enclosed in various joint modules. The manipulation of the gripper can be accomplished by altering the stiffness of each joint module separately. This principle of action is commonly used in multi-joint manipulator claws, as shown in Figure 12. 

Jing Taitan et al. of the Anhui University of Technology designed a passive adaptive robotic dexterous hand based on MRF, as shown in Figure 12a [97]. This dexterous hand can passively adjust the position of the contact point between the finger and the object according to the shape of the target object using the reaction force between the finger and the object during grasping and using the magnetorheological effect to lock the spring to achieve the envelope grasping of the object. By extending the joint module to two fingers, three fingers, or even more fingers, the grasping of objects of different sizes can be realized.

Kitano S. et al. of Kanazawa University, Japan, designed an MRG variable stiffness multi-joint manipulator for laparoscopic surgery, as shown in Figure 12b [26]. The variable stiffness manipulator consists of an MRG ring, electromagnets, a pull cord, and a magnetic spacer. The MRG ring is positioned between two electromagnets, with a magnetic spacer separating each adjacent electromagnet to prevent magnetic field leakage. When an external magnetic field is applied, the stiffness of the MRG increases, while it remains lower in the absence of a magnetic field. As a result, only the joint in the zero-field state bends when the pulling rope is pulled. The manipulation of different joints is achieved by selectively actuating specific pull cords, allowing for flexible control of the manipulator.

Leps T. et al. from the University of Maryland developed a low-power, blocking magnetorheological valve utilizing an electric permanent magnet, as illustrated in the left panel of Figure 12c [98]. This magnetorheological valve was integrated into a fluid-driven robot to enable the bending of different joints by controlling different valves. This design facilitated precise manipulation of the fluid robot, as depicted in the right figure of Figure 12c.

#### 3.2.2. The Magnet-Driven Type

The magnet-driven type grippers use MR materials subjected to forces in the presence of a magnetic field. These grippers achieve gripping and clamping directly through magnetic forces. The MR materials used for magnetic actuation are usually MREs and, rarely, MRFs.

**Figure 13:**
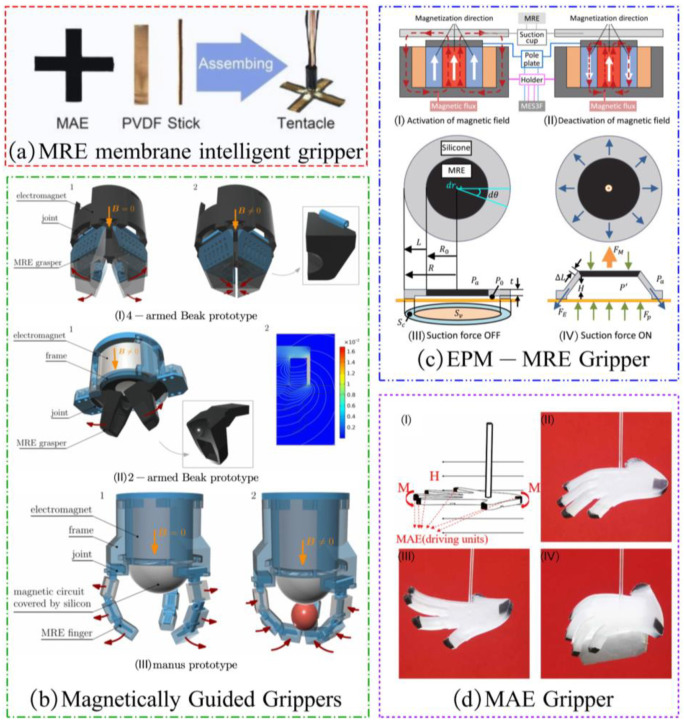
Examples of magnet-driven MRE grippers: (**a**) An MRE membrane intelligent gripper [100], (**b**) The magnet-driven grippers [29], (**c**) An EPM-MRE gripper [101], (**d**) An MAE gripper: (I) Driving mechanism of the soft gripper, and its three states, (II) resting, (II) opening, and (IV) gripping. [102].

Feng et al. of the University of Science and Technology of China developed an MRE membrane actuator by laminating a magnetorheological elastomer membrane (MRE membrane) with a polyvinylidene fluoride membrane (PVDF membrane) [99]. They created a magnet-driven type gripper using this actuator, as seen in Figure 13a [100]. The gripper has a deformation feedback function and can grasp and release objects under the control of a magnetic field.

Skfivan V. et al. developed three magnet-driven MRE grippers, as illustrated in Figure 13b [29]. The flexible gripper consists of MRE material wrapped around a rigid nonmagnetic skeleton. An electromagnetic coil is wound around a permanent magnet core. In the absence of current flowing through the coil, the elastomeric hand claw remains closed due to the influence of the magnetic field produced by the permanent magnet. When current is applied to the coil, the resulting electromagnetic field counteracts the external magnetic field, causing the elastomeric hand claw to open.

Zhang P et al. developed an electro-permanent magnet MRE (EPM-MRE) suction cup, as depicted in Figure 13c [101]. The deformation of the MRE suction cup was controlled by switching the poles of the electro-permanent magnet, enabling it to grasp objects. Li X et al. created a magnetically actuated elastomer (MAE) by combining silicone rubber with hard magnetic particles (NdFeB). And they applied this elastomer to fabricate a flexible gripper, as shown in Figure 13d [102]. This flexible gripper can be deformed to grasp objects through the influence of a magnetic field.

**Figure 14:**
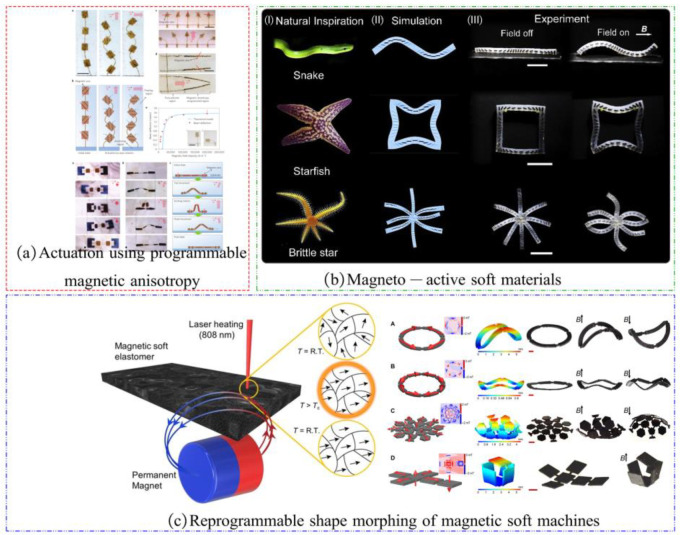
The magnet-driven soft robot: (**a**) Actuation using programmable magnetic anisotropy [103], (**b**) Magneto-active soft materials [104], (**c**) Reprogrammable shape morphing of magnetic soft machines [105].

In addition, magnetic actuation is also used in magnetic field-driven programmable soft robots, as shown in Figure 14. Usually, magnetic particles organized in a certain pattern are inserted in an elastic polymer to create magnetically actuated soft robots. This process “programs” the magnetic particles to meet specific scenario needs. When an external magnetic field is applied to the magnetically driven soft robot, the soft robot can be controlled to deform in a pre-programmed manner by controlling the strength and direction of the magnetic field, as shown in Figure 14a [103] and Figure 14b [104].

However, the magnetic field distribution inside this magnet-driven soft robot is designed and manufactured according to a predetermined pattern. Once completed, the internal magnetic field distribution is determined. Alapan Y et al. proposed a thermally assisted magnetic field programming method to reshape the internal magnetic field of the soft robot [105]. Permanent magnet particles embedded in an elastic polymer are heated. When the temperature exceeds the “Curie point”, a powerful magnetic field is delivered from the outside to alter how the internal magnetic field is distributed when the material cools. The team demonstrated a variety of structural models using a thermally assisted magnetization programming strategy, including a soft quadruped robot, a soft gripper, and a soft rolling ball, as shown in Figure 14c.

#### 3.2.3. The Adhesion Force Type

**Figure 15:**
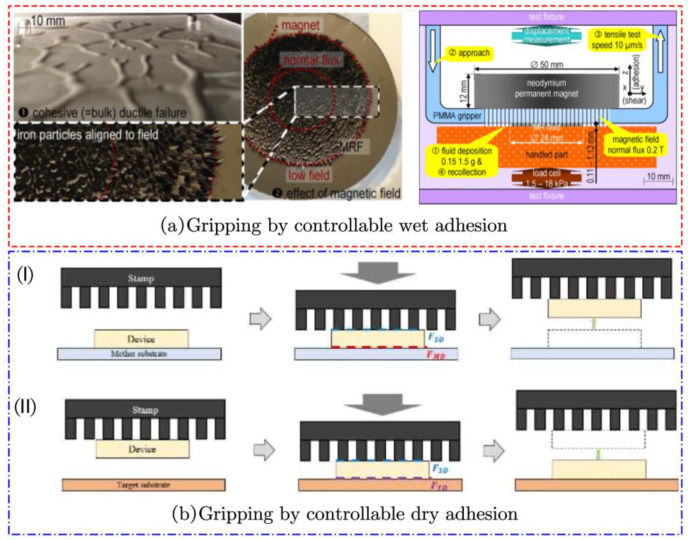
The adhesion force type: (**a**) Gripping by controllable wet adhesion [24]; (**b**) Gripping by controllable dry adhesion: (I) Picking process; (II) placing process. [106].

Adhesion can be classified into wet and dry adhesion based on the mechanism. Wet adhesion involves the insertion of a gel-like substance between the grasping surfaces, creating a liquid bridge, as observed in organisms such as snails. On the other hand, dry adhesion relies on the large contact area facilitated by van der Waals molecular forces and sub-microscopic surface weaving, enabling adhesion between contacting surfaces, as demonstrated by geckos. Inspired by these natural phenomena, researchers have successfully achieved flexible gripping through the implementation of wet and dry adhesion using MRF and MRE, respectively. This concept is illustrated in Figure 15.

Lanzetta M et al. designed a controlled wet adhesion robotic gripper using MRF as a medium, as shown in Figure 15a [24]. This flexible gripper consists of a polymer gripping surface and a permanent magnet. The MRF is applied to the polymer surface during gripping. By altering the magnetic field’s strength, the gripping surface and the item can attach and detach in different methods. It is also possible to control the adhesion strength, which can be applied to the adhesion of surfaces with different roughness and substrate materials.

Kim J H et al. fabricated an MRE hand claw through the process of photolithography. They utilized van der Waals forces for the chip transfer of micro LED, as illustrated in Figure 15b [106]. The relationship between the hardness and magnetorheological effects of MRE and carbonyl iron (CIP) content was also tested. Additionally, they performed experiments to obtain the correlation between adhesion force and magnetic field intensity.

## 4. Conclusions

This paper aims to introduce flexible robotic grippers, with a focus on grippers based on magnetorheological materials. It provides an insight into their distinctive characteristics and application by introducing common types of grippers, such as SMA, pneumatic flexible grippers, and dielectric elastomers. Additionally, the development and application prospects of MR materials are discussed. Subsequently, the magnetorheological flexible grippers are classified into three types: the viscosity/stiffness change type, the magnet-driven type, and the adhesion force type. We describe the advantages and drawbacks of these three types in terms of the most cutting-edge applications now used.

From the research in this work, we can conclude the following:

1. Flexible grippers have high adaptability and flexibility and satisfy the task of grip-ping objects of various shapes and materials. In medical applications, a 6 mm diameter pin SMA actuator can operate on a circular plane with a diameter of about 20 mm. In robotic applications, the pneumatic flexible gripper can realize the adaptive grasp of 564 g fruits with a diameter of about 100 mm. It is worth noting that the material and structure of the flexible gripper will change according to the weight and size of the grasping object.
2. The application of MR materials in flexible grippers has a broad development prospect. By controlling the strength of the magnetic field, the viscosity and stiffness of MR materials can be regulated, thus realizing flexible control of the grippers.
3. MR flexible grippers can achieve high precision and fast gripping action by magnetic drive. Due to the high response speed and good reversibility of MR material, the manipulator has high accuracy and stability in the gripping process.
4. Adhesive force is a crucial attribute of flexible grippers, and MR materials enable the regulation of adhesive force through control of the magnetic field distribution. Although the adhesive force is relatively small, this feature holds significant implications for handling objects with irregular shapes.
5. Flexible grippers based on MR materials have been proven usable and well-controllable by researchers. Next, MR flexible grippers should be developed in the direction of low power consumption and easy production. In the near future, it is believed that flexible grippers can achieve good applications in the industrial field.
